# CB_2_ Cannabinoid Receptors Contribute to Bacterial Invasion and Mortality in Polymicrobial Sepsis

**DOI:** 10.1371/journal.pone.0006409

**Published:** 2009-07-29

**Authors:** Balázs Csóka, Zoltán H. Németh, Partha Mukhopadhyay, Zoltán Spolarics, Mohanraj Rajesh, Stephanie Federici, Edwin A. Deitch, Sándor Bátkai, Pál Pacher, György Haskó

**Affiliations:** 1 Department of Surgery, University of Medicine and Dentistry of New Jersey-New Jersey Medical School, Newark, New Jersey, United States of America; 2 Department of Surgery, Morristown Memorial Hospital, Morristown, New Jersey, United States of America; 3 National Institute on Alcohol Abuse and Alcoholism, Bethesda, Maryland, United States of America; Johns Hopkins School of Medicine, United States of America

## Abstract

**Background:**

Sepsis is a major healthcare problem and current estimates suggest that the incidence of sepsis is approximately 750,000 annually. Sepsis is caused by an inability of the immune system to eliminate invading pathogens. It was recently proposed that endogenous mediators produced during sepsis can contribute to the immune dysfunction that is observed in sepsis. Endocannabinoids that are produced excessively in sepsis are potential factors leading to immune dysfunction, because they suppress immune cell function by binding to G-protein-coupled CB_2_ receptors on immune cells. Here we examined the role of CB_2_ receptors in regulating the host's response to sepsis.

**Methods and Findings:**

The role of CB_2_ receptors was studied by subjecting CB_2_ receptor wild-type and knockout mice to bacterial sepsis induced by cecal ligation and puncture. We report that CB_2_ receptor inactivation by knockout decreases sepsis-induced mortality, and bacterial translocation into the bloodstream of septic animals. Furthermore, CB_2_ receptor inactivation decreases kidney and muscle injury, suppresses splenic nuclear factor (NF)-κB activation, and diminishes the production of IL-10, IL-6 and MIP-2. Finally, CB_2_ receptor deficiency prevents apoptosis in lymphoid organs and augments the number of CD11b^+^ and CD19^+^ cells during CLP.

**Conclusions:**

Taken together, our results establish for the first time that CB_2_ receptors are important contributors to septic immune dysfunction and mortality, indicating that CB_2_ receptors may be therapeutically targeted for the benefit of patients suffering from sepsis.

## Introduction

Sepsis is defined as systemic illness caused by microbial invasion of normally sterile parts of the body. Sepsis is a major healthcare problem because its incidence is in the order of sepsis is in the order of 750,000 annually, and sepsis causes more than 200,000 deaths each year in the United States alone [1], [2]. Currently, therapeutic management of sepsis is limited mostly to supportive measures, in a large part due to a failure to fully establish the pathophysiology of this complex and heterogeneous syndrome. Multiple organ dysfunction syndrome and death in sepsis are consequences of an inability to kill invading pathogens effectively due to immunosuppression [3], [4]. Potentially contributing to immune suppression after a septic insult are immune cell apoptosis, inefficient phagocytosis of microbial pathogens by neutrophils and macrophages, decreased ability of antigen-presenting cells to present antigens, as well as decreased responsiveness of macrophages and T cells to release proinflammatory cytokines in conjunction with overzealous production of the anti-inflammatory cytokine IL-10 [5]–[7].

One view holds that immune dysfunction during sepsis is a result of autocrine/paracrine immunoregulatory mediators that are produced primarily at the site of infection/injury (including the bloodstream) and suppress immune cell function via acting on G-protein-coupled receptors. One group of these mediators are endocannabinoids, which elicit their cellular effects by binding to two subtypes of G-protein-coupled cannabinoid receptor proteins on the cell surface, termed CB_1_ and CB_2_ receptors [8]–[10]. Endocannabinoids are released from macrophages, dendritic cells, platelets, and parenchimal cells in response to inflammatory stimuli and oxidative stress [11]–[15] and are present at elevated concentrations in the sera of patients and animals suffering from septic, hemorrhagic or cardiogenic shock [16]–[20]. CB_2_ receptors are the dominant cannabinoid receptors on macrophages, neutrophils, and lymphocytes, and triggering CB_2_ receptors has an overall anti-inflammatory and immunosuppressive effect [21]. CB_2_ receptor activation augments the production of the anti-inflammatory cytokine, IL-10, by murine macrophages [22], and disrupts antigen processing by these cells, which leads to incomplete antigen-presentation to T cells [23], [24].

Despite the recent enormous advances in our knowledge of how CB_2_ receptors regulate immune function, the role of CB_2_ receptors in regulating bacterial sepsis is unknown. In the present study, using a genetic approach we examined the role of CB_2_ receptors in regulating the host's response to polymicrobial sepsis.

## Materials and Methods

### Experimental animals

CB_2_ knockout (KO) mice and their wild-type (WT) littermates were developed as described previously and had been backcrossed to a C57Bl/6J background [14]. All mice were maintained in accordance with the recommendations of the “Guide for the Care and Use of Laboratory Animals”, and the experiments were approved by the New Jersey Medical School Animal Care Committee.

### Cecal ligation and puncture (CLP)

Polymicrobial sepsis was induced by subjecting mice to CLP, as we have described previously [25], [26]. Eight- to twelve-week-old male CB_2_ KO or WT mice were anesthetized with Pentobarbital (50 mg/kg), given intraperitoneally (i.p.). Under aseptic conditions, a 2-cm midline laparotomy was performed to allow exposure of the cecum with adjoining intestine. Approximately two-thirds of the cecum was tightly ligated with a 3.0 silk suture, and the ligated part of the cecum was perforated twice (through and through) with a 20-gauge needle (BD Biosciences). The cecum was then gently squeezed to extrude a small amount of feces from the perforation sites. The cecum was then returned to the peritoneal cavity and the laparotomy was closed in two layers with 4.0 silk sutures. The mice were resuscitated with 1 ml of physiological saline injected subcutaneously (s.c.) and returned to their cages with free access to food and water. One group of mice was monitored daily and survival was recorded for 7 days. Another group of mice was reanesthetized with Pentobarbital (80 mg/kg i.p.) 16 hour after the operation, and blood, peritoneal lavage fluid, and various organs were harvested.

### Collection of blood, peritoneal lavage fluid, and organs

Blood samples were obtained aseptically by cardiac puncture using heparinized syringes after opening the chest and placed on ice into heparinized Eppendorf tubes until further processing for bacteriological analysis. After serial dilutions for bacteriological analysis were made (see below), the blood was centrifuged at 2,000×*g* for 10 min and the recovered plasma was stored at −70°C until further use. For peritoneal lavage, the abdominal skin was cleansed with 70% ethanol and the abdominal wall was exposed by opening the skin. Two milliliters of sterile physiological saline were then installed into the peritoneal cavity via an 18-gauge needle. The abdomen was massaged gently for 1 min while keeping the tip of the needle in the peritoneum, after which peritoneal fluid was recovered through the needle. Recovered peritoneal lavage fluid was placed on ice until processed for bacteriological examination. After serially diluting the peritoneal lavage fluid to determine colony forming unit (CFU) numbers (see below), the peritoneal lavage fluid was centrifuged at 5,000×*g* for 10 min and the supernatant was stored at −70°C until further analysis. Samples from spleen and thymus, were excised and immediately frozen in liquid nitrogen.

### Quantification of bacterial CFUs from peritoneal lavage fluid and blood

100 µl of blood or peritoneal lavage fluid was diluted serially in sterile physiological saline. 50 µl of each dilution was aseptically plated and cultured on trypticase blood agar plates (BD Biosciences) at 37°C. After 24 hours, the number of bacterial colonies was counted. Quantitative cultures are expressed as CFUs per milliliter of blood or peritoneal lavage fluid.

### Flow cytometric analysis of leukocyte subsets

Flow cytometric detection of leukocyte subsets was performed as previously described [27]. In brief, the percent distribution of leukocyte subsets in blood was analyzed by specifically staining CD3^+^ T-cells, CD19^+^ B-cells and CD11b^+^ myeloid cells using antibodies against CD markers conjugated with FITC, PERCP or PE (BD Biosciences) in three-color incubations. Aliquots of 0.1 ml whole blood were incubated with the respective markers for 15 min followed by incubation with BD FACS lysing solution (BD Biosciences) for 7 min at 37°C. Cells were washed twice with BD FACS wash buffer and then fixed with 1% methanol free formaldehyde. FACS acquisitions were performed in a centralized flow cytometry facility. At least 30,000 events were collected for each analysis.

### Protein extraction and Western blot analysis

Frozen organs were homogenized in a Dounce homogenizer in modified radioimmunoprecipitation assay buffer (50 mM Tris HCl, 150 mM NaCl, 1 mM EDTA, 0.25% sodium deoxycholate, 1% Nonidet P-40, 1 µg/ml pepstatin, 1 µg/ml leupeptin, 1 mM PMSF, 1 mM Na_3_VO_4_). The lysates were centrifuged at 15,000×*g* for 15 min, and the supernatant was recovered. Protein concentrations were determined using the Bio-Rad protein assay kit. A total of 40 µg of sample was separated on 8–12% Tris-glycine gel (Invitrogen Life Technologies) and transferred to nitrocellulose membrane. The membranes were probed with polyclonal rabbit anti-cleaved caspase-3, polyclonal rabbit anti-cleaved poly(ADP-ribose) polymerase (PARP), and polyclonal rabbit anti- inhibitory subunit of nuclear factor (NF)-κB (IκBα), (all from Cell Signaling Technology). Thereafter, the membranes were incubated with a secondary HRP-conjugated anti-rabbit antibody (Santa Cruz Biotechnology). HRP-conjugated polyclonal goat anti-β actin antibody to assess equal loading was used from Santa Cruz Biotechnology. Bands were detected using ECL Western Blotting Detection Reagent (Amersham Biosciences).

### Determination of lactate dehydrogenase (LDH), aspartate aminotransferase (AST), alanine aminotransferase (ALT), blood urea nitrogen (BUN), and creatine phosphokinase (CK) levels

Plasma concentrations of LDH, AST, ALT, BUN, and CK were analyzed using a clinical chemistry analyzer system (VetTest8008, IDEXX Laboratories).

### Determination of cytokine and chemokine levels

Concentrations of IL-10, IL-6, and MIP-2, were determined using commercially available ELISA kits (R&D Systems) according to the manufacturer's instructions. The lower detection limit for all these cytokines was 10 pg/ml.

### Statistical Analysis

Survival statistics were performed using Kaplan-Meier curve and log rank test. Two-tailed *t* testing was used to compare cytokine concentrations, CFUs, and other laboratory parameters. Statistical significance was assigned to *p* values smaller than 0.05.

## Results

### CB_2_ receptors contribute to sepsis-induced mortality and bacterial invasion

**Figure 1:**
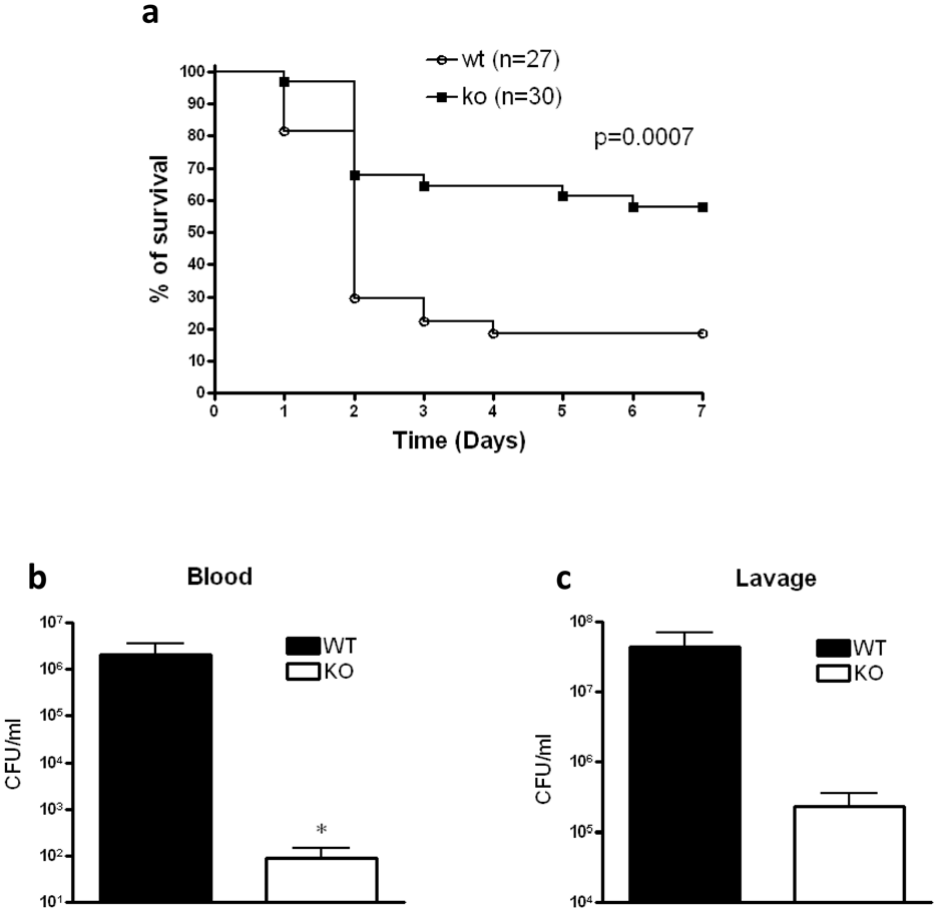
CB_2_ deficiency decreases mortality and bacterial burden in polymicrobial sepsis elicited by CLP. (a) Surviving CB_2_ KO and WT mice were counted every day for 7 days after inducing polymicrobial sepsis by way of cecal ligation and puncture (CLP). p<0.001 versus WT. (b) Blood and (c) peritoneal lavage fluid obtained from CB_2_ KO or WT mice 16 hour after CLP were cultured on soy-trypticase agar plates, and the number of bacterial colonies was counted. Data are the mean±SEM of *n* = 6–9 mice per group. *p<0.05.

To begin to study the role of CB_2_ receptors, we first investigated the effect of CB_2_ deficiency in CLP-induced septic peritonitis by monitoring the survival of CB_2_ WT and KO mice. As demonstrated in Figure 1a, CB_2_ receptor KO mice had significantly lower mortality rates compared with WT mice, which became apparent on the 2nd day of observation. On the 7th day following CLP, the mortality rate of CB_2_ KO mice was markedly (by more than 40%) lower than that of CB_2_ WT mice. No additional changes in mortality were detected when the mice were monitored for up to 10 days (data not shown). Because persistence of local bacterial infection and bloodstream invasion play important roles in mortality in the CLP model, we next assessed the impact of CB_2_ receptor inactivation on bacterial levels at the primary peritoneal site of infection and in the blood stream. We found markedly decreased numbers of bacteria in the blood but not peritoneal lavage fluid of CB_2_ receptor KO mice when compared to WT animals at 16 hours after CLP (Figure 1b, c).

Taken together, these studies document that CB_2_ receptors contribute to bacterial translocation into the bloodstream and mortality in polymicrobial sepsis.

### CB_2_ receptor inactivation diminishes the production of IL-10, IL-6 and MIP-2 in CLP-induced sepsis

**Figure 2:**
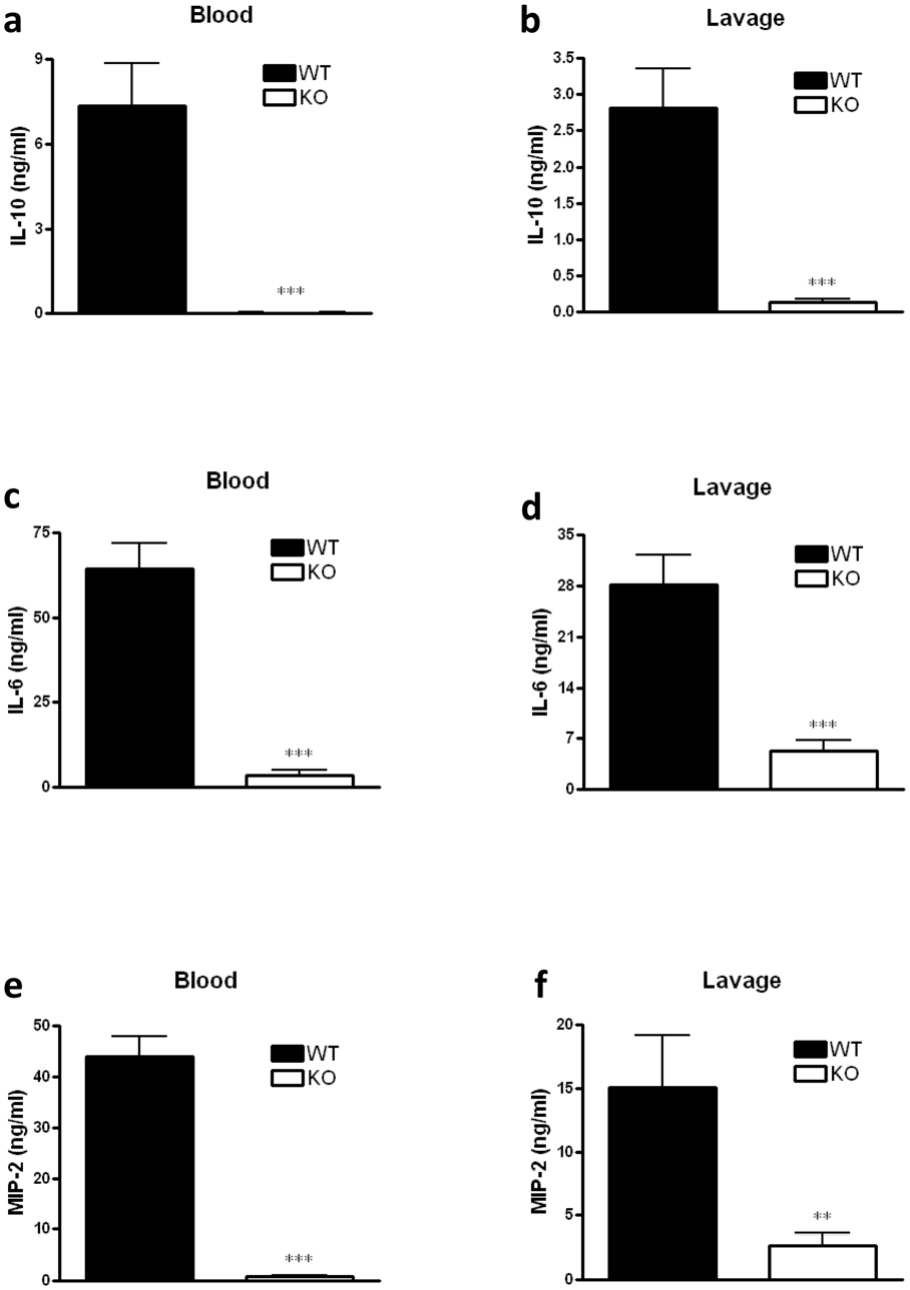
CB_2_ receptor deficiency decreases IL-10, IL-6, and MIP-2 levels in the plasma and peritoneal lavage fluid of mice subjected to CLP. IL-10 (a,b), IL-6 (c,d) and MIP-2 (e,f) concentrations were measured at 16 hours after surgery using ELISA. Data are the mean±SEM of *n* = 6–9 mice per group. ***p<0.001; **p<0.01.

Because IL-10 overproduction contributes to the impairment of host antibacterial defenses seen in mice undergoing CLP [28]–[31], we next compared IL-10 in the plasma and peritoneal lavage fluid of CB_2_ KO and WT mice subjected to CLP. CB_2_ KO mice exhibited markedly lower levels of IL-10 at 16 h after CLP (Figure 2a, b).

Because IL-6 blockade with neutralizing Abs has been shown to be protective in CLP-induced sepsis [32], we next assessed the role of CB_2_ receptors in regulating IL-6 production during sepsis. Plasma and peritoneal lavage fluid had lower levels of IL-6 in CLP-induced CB_2_ KO mice than their WT littermates (Figure 2c,d).

We then determined the levels of macrophage-inflammatory protein-2 (MIP-2), a crucial chemokine that mediates inflammatory responses, in the plasma and peritoneal lavage fluid of CB_2_ KO and WT mice subjected to CLP, and we found that CLP-induced concentrations of MIP-2 were diminished in CB_2_ KO mice as compared with their WT counterparts when measured at 16 h after CLP (Figure 2e, f).

### Mice deficient in CB_2_ receptor show decreased level of markers of tissue damage, and kidney and muscle injury

**Figure 3:**
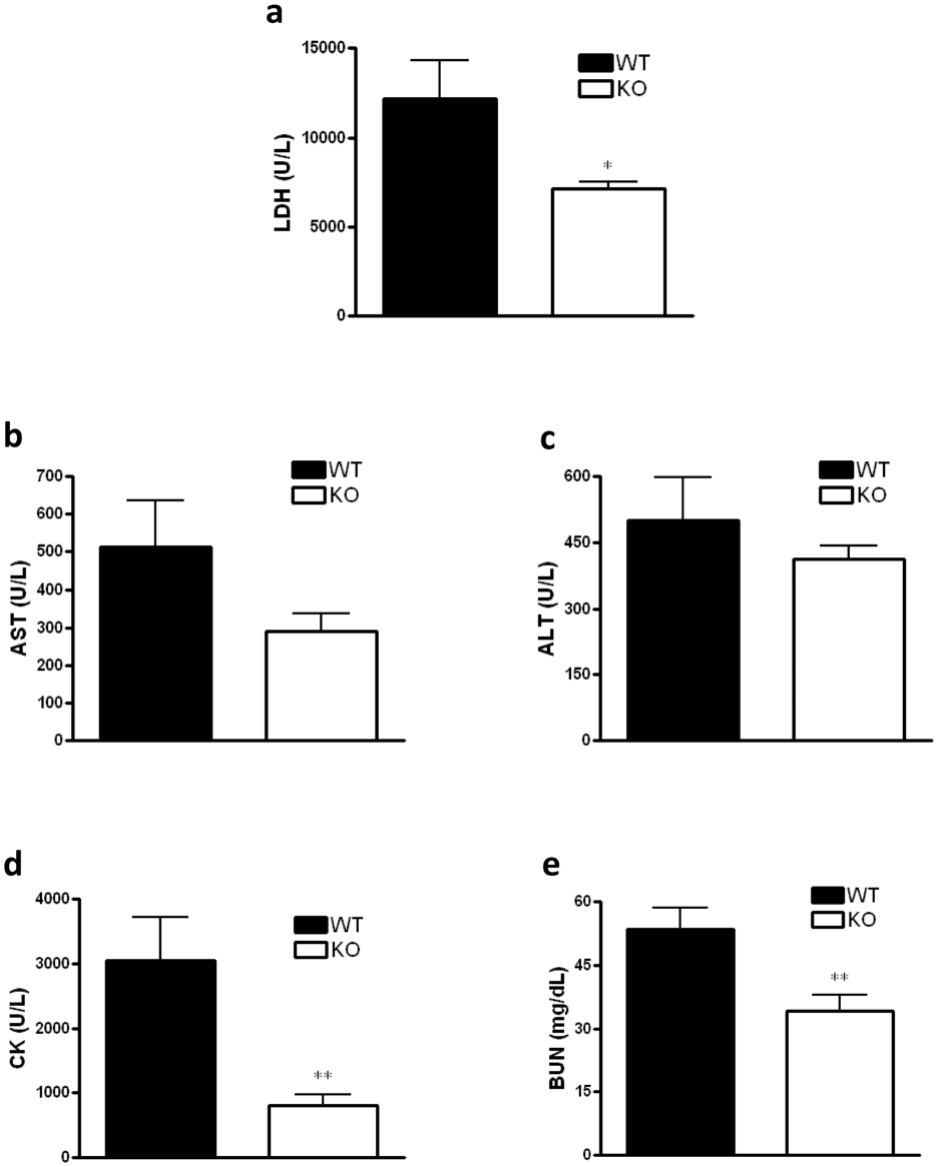
CB_2_ KO mice have less tissue damage, kidney and muscle injury in sepsis than their WT counterparts. LDH (a), AST (b), ALT (c), and CK (d) activity, and BUN (e) levels were measured in plasma samples 16 hour after CLP using a clinical chemistry analyzer system (VetTest8008, IDEXX Laboratories). Data are the mean±SEM of *n* = 6–9 mice per group. **p<0.01; * *p*<0.05.

We next measured markers of disease severity and organ damage in an attempt to provide further explanation for the improved survival of CB_2_ receptor KO mice. Levels of LDH (Figure 3a), were lower in CB_2_ KO mice, indicating less tissue damage in general. Markers of liver (AST and ALT) function were not different between the WT and KO groups (Figure 3b, c). In addition, we could not detect any lung inflammation in CLP-challenged mice as assessed by histological analysis of lung sections, and detecting myeloperoxidase activity in lung tissue homogenates after 16 hour of CLP (data not shown). Finally, CK activity and BUN levels were lower in CB_2_ KO mice indicating preserved kidney function and lessened muscle (both heart and skeletal) damage, respectively (Figure 3d,e).

### CB_2_ receptor deficiency decreases NF-κB activation in CLP-induced sepsis

**Figure 4:**
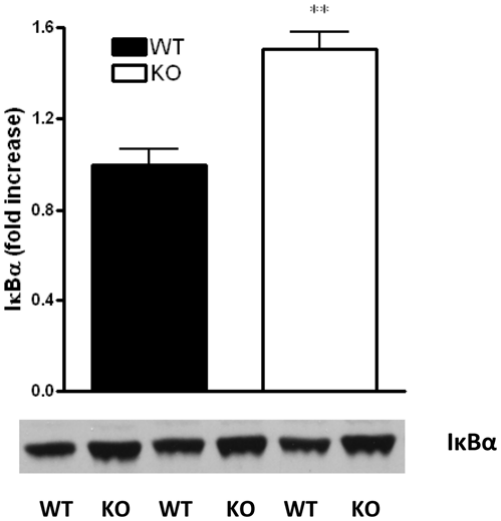
CB_2_ receptor deficiency is associated with augmented IκBα levels in CLP-induced sepsis. IκBα degradation was assessed using Western blotting of spleen protein extracts of CB_2_ WT and KO mice. Protein extracts were generated from spleen taken 16 hours after sepsis induction. Bands were detected by enchanced chemiluminescence (ECL). Results (mean±SEM) shown are representative of 3 experiments. **p<0.01 versus WT.

Microbial components and endogenous danger signals trigger the activation of signaling cascades leading to induction of the NF-κB system during sepsis. Persistent activation of NF-κB may cause excessive inflammatory responses culminating in tissue injury, organ dysfunction, and death. We, therefore, studied the activation of NF-κB by measuring levels of the IκBα in spleen of septic animals. As Figure 4 shows, the levels of IκBα were increased in the spleen of CB_2_ KO as compared to WT mice, indicating decreased NF-κB activation in KO mice.

### Genetic deletion of the CB_2_ receptor diminishes apoptosis in lymphoid organs

**Figure 5:**
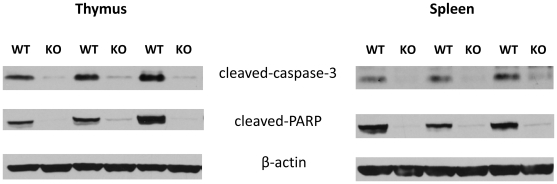
CB_2_ receptor deficiency decreases apoptosis in septic thymus and spleen. Proteolytic cleavage of caspase-3 and PARP in thymus and spleen protein extracts from CB_2_ WT and KO mice was examined using Western blotting. Bands were detected by enchanced chemiluminescence (ECL). Results shown are representative of 3 experiments.

Sepsis provokes extensive immune cell apoptosis that contributes to immune dysregulation and mortality. This was borne out by studies demonstrating that transfer of apoptotic splenocytes worsens survival in CLP-induced sepsis [33]. Because proteolytic cleavage of caspase-3 and PARP is a good indicator of apoptosis, we tested whether CB_2_ receptor deficiency would affect the cleavage of caspase-3 and PARP in the spleen and thymus of mice subjected to CLP. Figure 5 shows that 16 hours after the onset of sepsis, the cleavage of both caspase-3 and PARP was markedly decreased in thymus and spleen of CB_2_ receptor KO mice.

### Lack of CB_2_ receptors augments the number of CD11b^+^ and CD19^+^ cells during CLP

**Figure 6:**
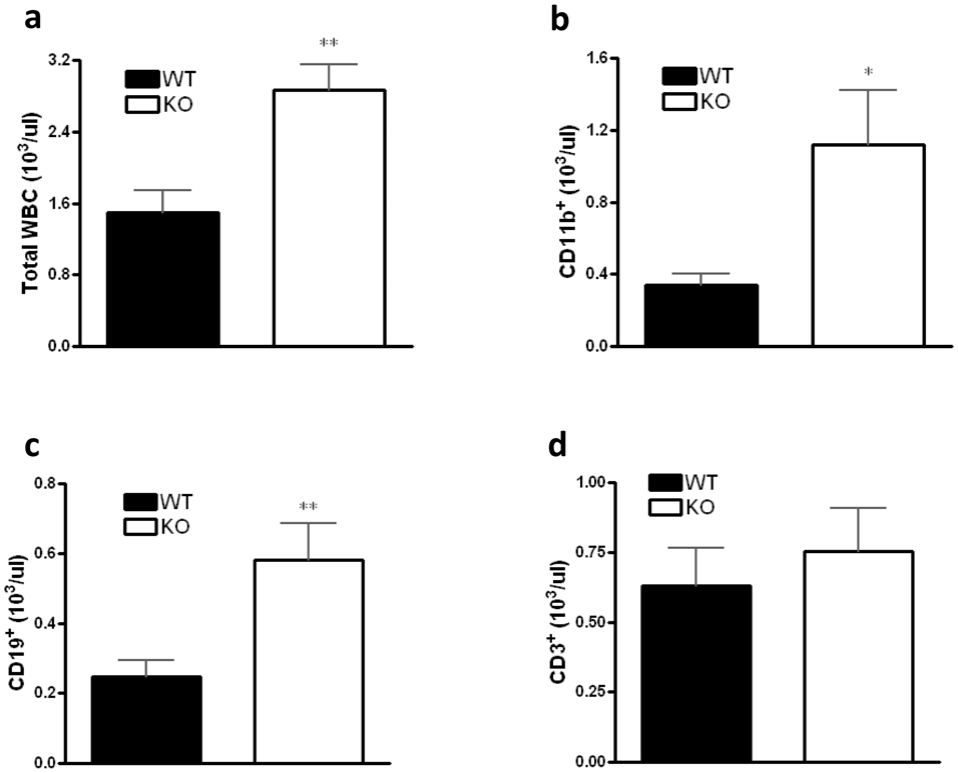
Effect of CB_2_ receptor inactivation on the leukocyte cell subsets in the blood during CLP. Numbers of total white cell (a), as well as CD11b^+^ (b), CD19^+^ (c), and CD3^+^ (d) cells were monitored in blood by flow cytometric analysis after 16 hours of sepsis induced by CLP. Data are the mean±SEM of *n* = 6–9 mice per group. *p<0.05; **p<0.01.

CLP-challenged mice exhibit a decrease in white blood cell numbers, which includes CD11b^+^ cells (mostly neutrophils), CD3^+^ T lymphocytes, and CD19^+^ B lymphocytes [34], [35]. Unchallenged CB_2_ KO mice have cell counts comparable to their WT counterparts [36]. Flow-cytometric analysis of cell counts revealed that CLP-challenged CB_2_ KO mice have increased numbers of total white blood cells (Figure 6a), CD11b^+^ (Figure 6b) cells and CD19^+^ B lymphocytes (Figure 6c) in comparison with CB_2_ WT mice, whereas the number of CD3^+^ T cells (Figure 6d) was comparable between WT and KO mice undergoing CLP. We propose that preserved white blood cell numbers in KO animals contribute to the decreased bacterial growth and mortality of these mice.

## Discussion

Current concepts suggest that sepsis is the consequence of the inability of the immune system to ward off infecting pathogens due to immune system dysfunction. The mechanisms underlying these immune functional abnormalities are largely unknown. Recent studies have expanded the list of potential mediators to molecules that are produced locally by infected and inflamed tissues and act on specific G protein-coupled receptors expressed on immune cells to inhibit their function. These molecules include adenosine [37], [38], which bind to and trigger their receptors on lymphocytes, macrophages, and neutrophils, thereby diminishing anti-bacterial defenses. In these studies we have focused on a new type of immunosuppressive G protein-coupled receptor, the CB_2_ cannabinoid receptor, which is expressed primarily by immune cells and is activated by locally released endocannabinoids. Using the CLP model of sepsis, we found that CB_2_ receptor activation by endogenously released cannabinoids contributes to mortality, bacterial invasion, IL-10 production, and immune cell death in sepsis.

Studies utilizing antibiotic therapy have shown that systemic bacterial dissemination is a major factor contributing to the mortality of both experimental animals and humans during sepsis [39]–[41]. Our studies showed that bacterial burden was decreased in CB_2_ receptor KO mice suggesting that CB_2_ receptor activation contributes mortality by increasing systemic bacterial burden during sepsis. One potential explanation for the decreased bacterial load in mice lacking CB_2_ receptors is a decrease in the levels of the immunosuppressive IL-10 leading to a better preserved phagocytic response. IL-10 is an immunoregulatory cytokine that is released primarily by macrophages during sepsis. IL-10 is an important contributor to the dysregulated immune system that is observed in sepsis [28]–[31], [42]. Recent studies have shown that CB_2_ receptor activation can upregulate IL-10 production across a number of experimental systems that utilize macrophages [22], [43]. Given the relevance of IL-10 in sepsis, the effect of CB_2_ receptor activation on IL-10 release is likely to be a major determinant of the immunomodulatory action of CB_2_ receptor activation in sepsis.

Sepsis instigates widespread immune cell apoptosis, and mortality in this illness is thought to be, at least in part, a consequence of dysregulated immune cell death [44]–[46]. CB_2_ receptor triggering induces both T and B lymphocyte apoptosis in vitro [47]–[52]. Our data showing decreased levels of caspase-3 cleavage as well as PARP cleavage in CB_2_ KO mice following sepsis indicate that CB_2_ receptors are essential contributors to apoptotic processes also in vivo. Moreover, we found increased numbers of CD11b^+^ and CD19^+^cells in blood of CB_2_ KO mice suggesting that CB_2_ receptors contribute to bacterial invasion by CD11b^+^ and B cell depletion in the bloodstream. The immunosuppressive role of CB_2_ receptors has been confirmed recently in vivo by inducing immune-mediated inflammatory disease in CB_2_ receptor KO and WT mice. CB_2_ receptor KO mice develop a more severe form of experimental allergic encephalomyelitis in comparison with WT mice, which is a consequence of increased T-cell activation and decreased T-cell apoptosis in KO vs. WT mice [53]. In another model, CB_2_ receptor KO mice displayed increased allergic responses in the skin, which was secondary to increased production of proinflammatory cytokines [54]. Moreover, we have recently demonstrated that CB_2_ receptor KO mice exhibit exacerbated liver injury following hepatic ischemia/reperfusion, which is associated with increased production of pro-inflammatory cytokines and neutrophil infiltration into the liver [14]. Our results that CB_2_ receptor inactivation decreases mortality during CLP-induced bacterial (non-sterile) sepsis might seem contradictory to the observations in experimental allergic encephalomyelitis, allergic skin inflammation, and ischemia/reperfusion-induced inflammation in that CB_2_ receptor inactivation is injurious in these sterile inflammation models. But we believe the data seen as a whole suggests that differences in outcome in the two types of model are due to immunosuppression being beneficial in sterile inflammation/ischemia but detrimental in clinically relevant models of infection-induced sepsis where mortality depends more upon the inability to mount an immune response leading to a loss of control of bacterial growth.

In summary, we found that CB_2_ receptor activation by endogenously released cannabinoids contributes to mortality, bacterial invasion, IL-10 production, and immune cell death in sepsis. Based on these observations, we suggest that CB_2_ receptor triggering contributes to the development of immune system dysfunction that leads to mortality in sepsis.
